# Artificial neural network-augmented dosiomic integration for predicting distant recurrence in NSCLC patients treated with SBRT

**DOI:** 10.3389/fonc.2025.1669954

**Published:** 2025-09-25

**Authors:** Kaushik Halder, Ryan Alden, Rihan Podder, M. Felix Orlando, Michael D. Mix, Tithi Biswas, Jeffrey B. Bogart, Tarun K. Podder

**Affiliations:** ^1^ Department of Radiation Oncology, SUNY Upstate Medical University, Syracuse, NY, United States; ^2^ Department of Electrical Engineering, Indian Institute of Technology Roorkee, Roorkee, India; ^3^ Department of Radiation Oncology, University of Florida, Gainesville, FL, United States

**Keywords:** artificial neural network, machine learning, treatment response modeling, SBRT, NSCLC, distant recurrence

## Abstract

**Objective:**

Stereotactic body radiotherapy (SBRT) is a standard curative treatment for inoperable early-stage non-small cell lung cancer (NSCLC) patients. However, the high rate of distant recurrence following radiotherapy remains a significant clinical challenge. This study focuses on developing a machine learning model for distant recurrence prediction using diverse dosiomic and patient-specific clinical features. The proposed model aims to assist clinicians in informed decision-making, individualized treatment decisions to improve post-SBRT outcomes.

**Method:**

This study utilized a multi-institutional dataset comprising 575 NSCLC patients who underwent SBRT. A total of 21 features, comprising 14 dosimetric and 7 clinical variables, were incorporated for developing the predictive framework. The predictive model was developed based on an artificial neural network (ANN) architecture with several dense layers. Model training and internal validation were conducted using data obtained from one institution, while external validation was performed utilizing data from an independent institution. To enhance clinical interpretability, SHAP analysis was employed to evaluate the relative importance of each feature contributing to the model’s output.

**Results:**

The initial predictive model, developed using individual clinical and dosimetric features, achieved area under the receiver operating characteristic curve (ROC-AUC) in the range of 0.64 to 0.65 while validated with an external dataset, respectively. To enhance predictive performance, dosimetric features were integrated with clinical variables, resulting in improved ROC-AUC values of 0.75 for internal validation with 10-fold cross validation technique and 0.71 for external validation with 1000 bootstrap iterations. Dosiomic features enhanced performance by 9-11%, highlighting their importance in distant recurrence prediction. Additionally, to enhance the interpretability of the model’s predictions, SHAP-based analysis was conducted, revealing that the number of treatment fractions, dose per fraction, and minimum dose to GTV were among the five most influential dosiomic features.

**Conclusion:**

This study introduces an ANN-based model for predicting distant recurrence in NSCLC patients followed by SBRT. This study also demonstrates the impactful dosimetric and clinical features for the designed predictive model, highlighting its potential as an assistive tool for informed and individualized treatment planning in clinical practice.

## 1 Introduction

Lung cancer remains the foremost cause of cancer-related mortality, with projections indicating that over 227,000 individuals will be diagnosed with the disease by 2025 (1). Among these cases, approximately 80%-85% are expected to be classified as non-small cell lung cancer (NSCLC) (2–5). Stereotactic body radiotherapy (SBRT) is recognized as one of the standard curative treatment modalities for patients with early-stage NSCLC. A primary advantage of SBRT lies in its ability to deliver high-dose radiation precisely to the tumor site over a limited number of treatment fractions, thereby achieving a substantial biologically effective dose. Clinical guidelines recommend the use of SBRT primarily for patients who are medically unsuitable for surgical intervention due to advanced age, compromised health status, or an inability to accept the potential risks associated with conventional surgery (6). Although dose-escalation studies in SBRT have demonstrated enhanced patient outcomes, with improved local recurrence control and overall survival rates, a significant proportion of patients continue to develop distant recurrence, contributing to elevated mortality risks (7–9). Therefore, there is a critical need for accurate and noninvasive prediction of distant recurrence prior to SBRT, assisting clinicians to tailor treatment strategies based on individual patient risk profiles and optimize therapeutic decision-making.

Treatment response predictive modeling is a crucial objective in radiation therapy, enabling the correlation between treatment data and clinical outcomes (10–15). Radiomics and imaging feature-based machine learning models have recently advanced in predicting post-treatment recurrence across various therapeutic modalities (16–23). Nemoto et al. (21) demonstrated the efficacy of machine learning-based recurrence (local, regional, and distant recurrences) prediction models using Positron Emission Tomography (PET) and Computed Tomography (CT) -based radiomics features extracted from the 82 NSCLC patients treated with SBRT. Furthermore, Kakino et al. (22) enhanced the model’s performance to predict local and distant recurrences while integrating clinical data and breath-hold CT-based clinical features collected from a multi-institute SBRT cohort. However, the performance of radiomics-based models is highly influenced by the quality of the imaging data; suboptimal image quality can result in erroneous feature extraction, ultimately impairing the accuracy of treatment response predictions.

Moreover, in the existing literature, only a limited number of studies (24, 25) have developed recurrence prediction models using a small set of dosiomic features along with patient characteristics and clinical data. Mohamed et al. (24) developed a recurrence prediction model while incorporating a comprehensive set of features, including diagnostic parameters (tumor classification and histology), patient demographics, comorbidities, and detailed treatment (chemotherapy, radiotherapy, and surgery) records. Similarly, Hindocha et al. (25) incorporated a wide range of patient-specific clinical variables along with radiation dose, number of fractions, and planning target volume as well as types of treatment modalities, to model recurrence and recurrence-free survival. However, these studies (24, 25) lack specification of predicted recurrence subtype and encompass heterogeneous treatment modalities.

Therefore, the development of distant recurrence prediction models for early-stage NSCLC patients treated with exclusively SBRT remains limited. To address this research gap, this study especially focuses on the development and validation of an Artificial Neural Network (ANN)-based distant recurrence prediction model for early-stage NSCLC patients treated with SBRT. A key innovation of this work lies in the integration of diverse dosimetric parameters from radiotherapy planning alongside routinely available patient-specific clinical features. Dosimetric features offer significant advantages by being directly associated with treatment planning parameters and exhibiting reduced dependence on imaging quality. This direct linkage enables dosiomic models to effectively capture the influence of dose heterogeneity on therapeutic outcomes. Additionally, as dosiomic features are extracted from radiation treatment plans, they inherently benefit from higher levels of standardization and reproducibility, minimizing the variability often introduced by differences in imaging protocols and acquisition systems. By representing the actual therapeutic intervention rather than anatomical characteristics alone, dosiomic-based models can enhance the precision and reliability of outcome prediction. This approach provides deeper insights into individual patient responses and potential treatment-related toxicities, ultimately supporting more personalized and effective radiation therapy strategies. Another important aspect of this study is the emphasis on enhancing model’s interpretability by evaluating the impact of each predictive feature, thereby facilitating easier understanding and integrating the model’s insights into clinical decision-making processes. The major contributions of this work are outlined below:

1. Design and development of an ANN-based distant recurrence prediction model for early-stage NSCLC patients treated with SBRT.
2. This study incorporates two institutes’ data (Dataset-A: n=370; Dataset-B: n=205) for training and testing of our designed model. A key strength of the methodology lies in the use of Dataset-A for internal training and validation through a 10-fold cross validation strategy. In parallel, Dataset-B serves as an independent cohort for external validation, implemented using 1000 bootstrap iterations.
3. In the field of machine learning-based treatment response prediction models, limited information is often available regarding the extent to which features contribute to model prediction. To address this limitation, this study presents a SHapley Additive exPlanations (SHAP)-based feature importance analysis while providing enhanced interpretability of the designed distant recurrence prediction model.

## 2 Methods and materials

### 2.1 Patient’s demographic and treatment characteristics

Data were collected from 575 patients diagnosed with early-stage NSCLC who underwent SBRT between 2013 and 2022 at two distinct healthcare institutions. The distribution of disease stages at the time of treatment revealed that the majority (85.4%) of patients presented with stage I lung cancer, while 14.6% were classified as stage II, whereas this study excluded advanced-stage disease (stage III or IV). As per two institute datasets, distant recurrence was observed in 84 patients within 5 years completing SBRT. This study cohort consisted of 50.6% male and 49.4% female patients with the median age of 75 years (range: 42–98 years). Following SBRT, patients underwent clinical follow-up every three months during the first two years. Between years three and five post-treatment, evaluations were conducted at six-month intervals, transitioning to annual assessments thereafter. SBRT for early-stage NSCLC in this cohort was administered using various dose-fractionation treatment parameters: 48 Gy in 3 fractions (n = 1), 48 Gy in 4 fractions (n = 7), 50 Gy in 4 fractions (n = 120), 50 Gy in 5 fractions (n = 244), 54 Gy in 3 fractions (n = 60), 55 Gy in 5 fractions (n = 18), 57 Gy in 4 fractions (n = 1), 60 Gy in 3 fractions (n = 1), and 60 Gy in 5 fractions (n = 123). In the present analysis, the prescribed radiation doses for each patient yielded a biologically effective dose (BED), calculated with an $\frac{\alpha }{\beta }\;$ ratio of 10, ranging from 100 Gy to 180 Gy, with the criterion that at least 95% of the planning target volume received the prescribed dose. The most frequently utilized prescription dose was 50 Gy administered over 5 fractions, corresponding to a BED of 100 Gy.

### 2.2 Study dataset

In this study, datasets from two independent institutions (IRB No. xxxx and xxxx) were incorporated to enhance the robustness and generalizability of the designed prediction framework. The dataset from the first institute, referred as Dataset-A that comprised 370 early-stage NSCLC patients’ records, which was utilized for the predictive model’s training and validation. Concurrently, an independent dataset from the second institute, designated as Dataset-B that included 205 patients, which was employed for the model’s external validation purpose to assess the robustness of the model. A comprehensive overview of the characteristics of both datasets is presented in **Table 1**. Each dataset encompassed a total of twenty-one variables, consisting of 7 patient specific relevant clinical features and 14 important dosiomic inputs. The 7 clinical features were Age (years), Tumor lobe, Age adjusted Charlson comorbidity index (aCCI), PET Max SUV, T-stage, Histology, and Smoking pack per year. Fourteen dosimetric features were planning target volume (PTV) (cc), gross tumor volume (GTV) (cc), Total lung volume (cc), Prescription dose (Gy), Number of fractions, Dose per fraction (Gy), Minimum dose to PTV (Gy), Maximum dose to PTV (Gy), Median dose to PTV (Gy), Minimum dose to GTV (Gy), Maximum dose to GTV (Gy), Mean dose to GTV (Gy), Mean dose to lung (Gy) (average dose across bilateral lung contours excluding GTV), and Conformity index. In addition, Spearman’s rank correlation analysis was conducted using a two-tailed test with a 95% confidence interval to determine the significance with the study endpoint, as reflected by the corresponding p-values (given in **Table 1**). The details regarding the dosiomic feature extraction procedure are provided in the **Supplementary File**. The distribution of features across the two datasets was analyzed and is presented in **Supplementary Figure S2** (in **Supplementary File**).

**Table 1:** Data cohort of 575 early-stage NSCLC patients: description of dosimetric and clinical features.

Feature groups	Feature variables		Data-cohort: A (n=370) median (range)/(%)	P-value	Data-cohort: B (n=205) median (range)/(%)	P-value
Clinical numerical features	Age (yr)		75 (49-92)	0.65	74 (42-98)	0.96
	Smoking Pack years		50 (0-150)	0.23	40 (0-145)	0.06
	PET Max SUV		5.5 (0.8-37.4)	0.73	5.97 (0.84-35)	0.39
	aCCI		8 (3-14)	0.99	7 (2-13)	0.04
Clinical categorical features	Tumor Lobe:	Upper	65.1%	0.84	70.73%	0.99
		Middle	6.5%		14.15%	
		Lower	28.4%		15.12%	
	T-stage:	1a	11.6%	0.55	58.54%	0.88
		1b	51.1%		26.34%	
		1c	23%		0%	
		2a	11.9%		13.17%	
		2b	2.4%		1.95%	
	Histology: Squamous cell carcinoma		42.4%	0.94	62.93%	0.79
	Non-small cell carcinoma		8.4%		0%	
	Adenocarcinoma		31.1%		26.83%	
	Others		18.1%		10.24%	
Dosimetric parameters	Conformity index		1.12 (0.92-2.86)	0.44	1.04 (0.58-1.7)	0.76
	PTV volume (cc)		34.83 (5.5-214.19)	0.74	20.2 (4.2-127.2)	0.47
	GTV volume (cc)		10.64 (0.63-126)	0.46	5.1 (0.4-67.3)	0.49
	Total lung volume (cc)		3808.75 (1472.48-9509.5)	0.83	3411.3 (1297.8-6875.3)	0.26
	Prescription dose (Gy)		50 (48-60)	0.01	60 (48-60)	0.83
	Dose per fraction (Gy)		10 (10-20)	0.007	12 (9-27)	0.42
	Number of fractions		5 (3-5)	0.002	5 (3-5)	0.34
	Minimum dose to GTV (Gy)		53.45 (30.58-70.11)	0.04	60.06 (42.61-71.27)	0.67
	Maximum dose to GTV (Gy)		65.38 (31.18-82.75)	0.05	66.55 (50.67-84.53)	0.69
	Mean dose to GTV (Gy)		60.10 (6.21-72.31)	0.07	63.47 (50.03-77.18)	0.65
	Minimum dose to PTV (Gy)		45.49 (21.7-56.90)	0.04	51.85 (29.63-59.84)	0.88
	Maximum dose to PTV (Gy)		65.61 (6.47-83.92)	0.50	67.11 (51.21-84.53)	0.70
	Mean dose to PTV (Gy)		56.90 (6.11-67.42)	0.01	61.62 (48.81-72.53)	0.68
	Mean lung dose (Gy)		3.82 (0.66-21.86)	0.90	2.92 (0.91-25.6)	0.005

### 2.3 Development of distant recurrence prediction model

**Figure 1:**
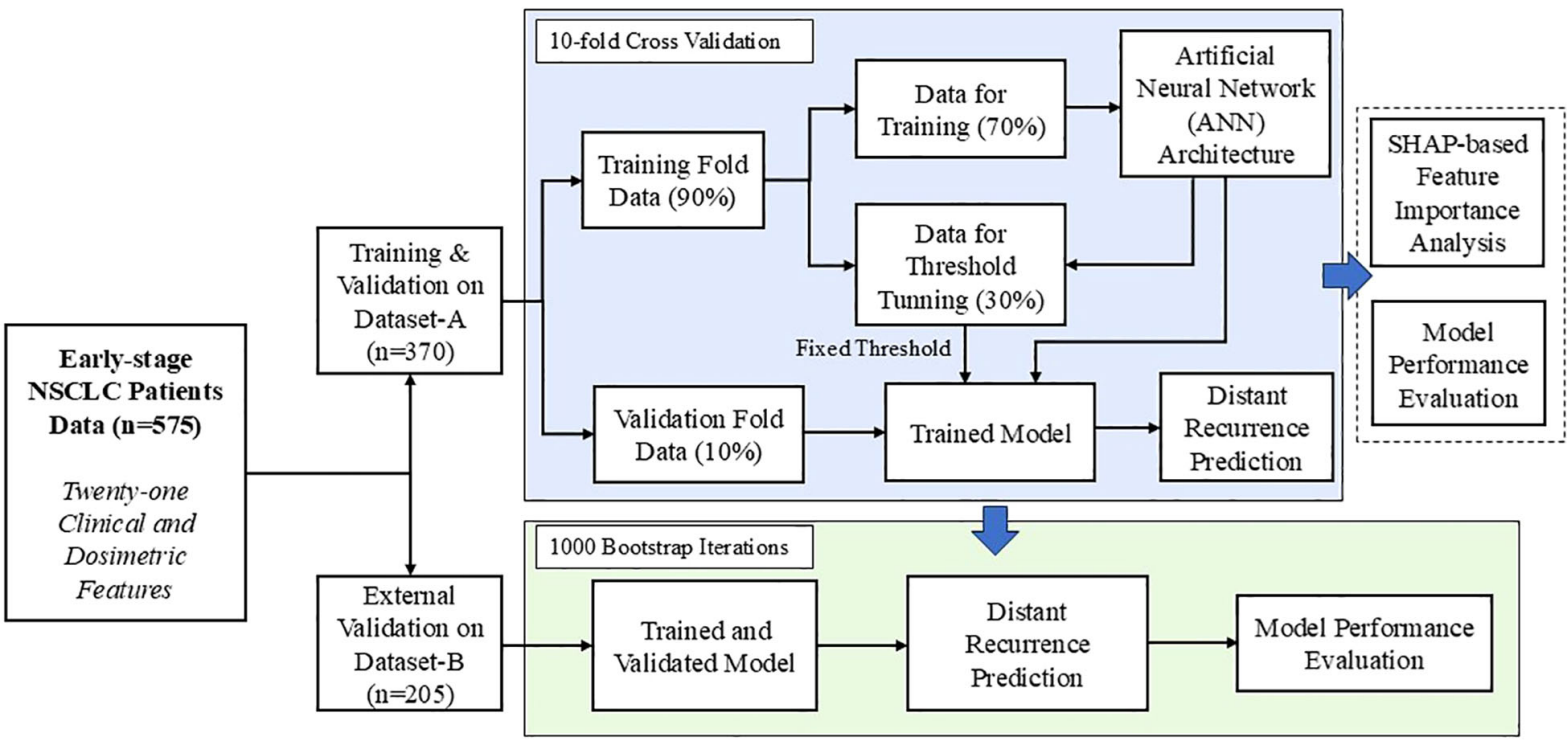
Schematic representation of the proposed machine learning-based framework for distant recurrence prediction with model design and validation workflow.

A major contribution of this study lies in the development of a distant recurrence prediction model utilizing ANN framework, while deploying a comprehensive set of twenty-one clinical and dosimetric features. The constructed feedforward neural network architecture comprised with several fully connected hidden layers and the pipeline of the developed model is given in **Supplementary File** (**Supplementary Figure S1**). Each hidden layer employed the Rectified Linear Unit (ReLU) as an activation function, whereas the output layer utilized a sigmoid activation function to facilitate binary classification. The output layer of the proposed ANN model was configured to classify early-stage NSCLC patients into two outcome categories: distant recurrence and non-recurrence. In this study, a ten-fold stratified cross-validation procedure was applied to the internal dataset (n = 370). For each fold, 90% of the data were allocated for model development, while the remaining 10% were reserved as an independent holdout set with a positive incident rate of 18%. Within the 90% subset, data were further partitioned into 70% for training and 30% for validation. The threshold was optimized during this validation phase using the F1-score as the performance criterion. The F1-score, derived from the harmonic mean of precision and recall using Precision-Recall (PR) curve. Once the optimal threshold was obtained, it was fixed and subsequently employed for evaluation on both the holdout portion of the internal dataset and an external independent dataset from a separate institution (as presented in the **Supplementary File**: Pseudo code). Overall schematic representation of the model architecture and the validation workflow is illustrated in **Figure 1**. The supervised ANN-based model was trained using the Adaptive Moment Estimation (Adam) optimizer (26). To mitigate overfitting, L2 regularization with a penalty coefficient of 0.01 was applied to the kernel weights of each hidden layer. Furthermore, batch normalization was incorporated to each hidden layer, thereby mitigating the challenges associated with vanishing and exploding gradients. An early stopping criterion was also employed to stop the training when no further improvement in the model’s performance on the validation set was observed, thereby avoiding unnecessary iterations and potential overfitting. The detailed description of the designed ANN-based prediction model, including pseudo code, is provided in **Supplementary File**. In this study, class imbalance is a significant concern in constructing reliable models for predicting treatment response. To address this issue, two strategies were employed: class weighting and focal loss. The class weighting scheme was introduced to assign higher penalties to misclassifications of the minority class, thereby improving its representation during training. In parallel, focal loss was integrated into the optimization process to diminish the influence of easily classified majority samples, ensuring that the model emphasized harder-to-classify minority instances. The combined application of these methods effectively alleviated the adverse effects of class imbalance and enhanced predictive performance, particularly for the minority class.

## 3 Results

### 3.1 ANN-based distant recurrence predictive model’s performance

In this study, two different performance analyses were carried out to assess the efficacy and robustness of our designed prediction model. **Table 2** summarizes the evaluation results for distant recurrence prediction on Dataset-A, utilizing 10-fold cross validation technique. To further validate the generalizability and robustness of our designed predictive model, an external validation was performed using an independent dataset (Dataset-B) using 1000 bootstrap iterations and the corresponding performance analysis is presented in **Table 3**. **Tables 2** and **3** demonstrate that the model incorporating only seven clinical features achieved ROC-AUC of 0.69 and 0.65 for internal and external datasets, respectively. Evaluation with dosimetric features alone resulted the mean ROC-AUCs of 0.71 for Dataset-A and 0.64 for Dataset-B. Furthermore, to provide a more comprehensive assessment of performance evaluation, additional evaluation metrics were also considered, including Sensitivity, Specificity, Weighted average F1-score, Precision-Recall AUC (PR-AUC), Matthews correlation coefficient (MCC), and Positive predictive value. As established in prior literatures (27, 28), the assessment of predictive performance in the context of imbalanced datasets must be interpreted relative to the class distribution. Specifically, the baseline performance is determined by the prevalence of the positive (minority) class, which serves as the expected outcome under random prediction.

**Table 2:** Performance evaluation of the proposed distant recurrence-predictor model incorporating clinical and dosimetric features, assessed on Dataset-A using 10-fold cross validation.

Features	Performance metrics						
	ROC-AUC	Sensitivity	Specificity	Weighted average f1-score	Positive predictive value	MCC	PR-AUC
Clinical Features only	0.69 (0.09)	0.70 (0.19)	0.67 (0.21)	0.71 (0.14)	0.38 (0.17)	0.34 (0.14)	0.33 (0.14)
Dosimetric Features only	0.71 (0.06)	0.74 (0.18)	0.69 (0.21)	0.72 (0.13)	0.42 (0.21)	0.38 (0.14)	0.40 (0.13)
Dosimetric and Clinical Features	0.75 (0.04)	0.69 (0.21)	0.81 (0.16)	0.80 (0.08)	0.54 (0.20)	0.48 (0.09)	0.46 (0.09)

[mean (standard deviation)].

**Table 3:** Performance evaluation of the proposed distant recurrence-predictor model incorporating clinical and dosimetric features, assessed on external independent Dataset-B using 1000 bootstrap iterations.

Features	Performance metrics						
	ROC-AUC	Sensitivity	Specificity	Weighted average f1-score	Positive predictive value	MCC	PR-AUC
Clinical Features only	0.65 (0.53-0.80)	0.59 (0.20-1)	0.70 (0.08-1)	0.69 (0.17-0.88)	0.41 (0.18-1)	0.29 (0.08-0.60)	0.32 (0.15-0.60)
Dosimetric Features only	0.64 (0.56-0.77)	0.53 (0.14-0.88)	0.75 (0.40-1)	0.60 (0.26-0.82)	0.26 (0.19-0.47)	0.23 (0.10-0.46)	0.21 (0.14-0.39)
Dosimetric and Clinical Features	0.71 (0.65-0.82)	0.63 (0.39-0.98)	0.80 (0.40-1)	0.68 (0.47-0.89)	0.36 (0.23-0.86)	0.36 (0.25-0.61)	0.36 (0.20-0.62)

[mean (95% CI)].

Notably, the model developed with clinical and dosiomic feature individually demonstrated substantial performance based on positive predictive value, MCC, and PR-AUC metrics. Mostly, the lower bound of 95% confidence interval (CI) was below the prevalence level, indicating reduced prediction performance. However, our designed ANN-based model with the combination of dosimetric data with clinical features, demonstrated an improved performance as mean ROC-AUC of 0.75 (0.04) for the Dataset-A in 10-fold cross validation technique, while a comparable ROC-AUC score as 0.71(95% CI: 0.65-0.82) for external dataset. **Tables 2** and **3** present the detailed performance analysis of our designed model, indicating reliable classification performance across multiple evaluation metrics. The comprehensive performance analyses also highlighted that incorporating dosimetric features significantly improve the predictive capability of our designed ANN model, yielding ROC-AUC gains of 5.6-8.7% and 9.2-10.9% in ROC-AUC metric across the two datasets, respectively. Moreover, the improvement based on PR-AUC was observed as 15% relative to dosiomic feature based model and 39.4% relative to clinical feature-based model for Dataset-A. Specifically, the mean PR-AUC increased with the lift (performance metric/model’s prevalence) range of 2.11 to 2.55 from the baseline, indicating a meaningful improvement in model performance (27). Similarly, the model yielded the MCC score of 0.48 for Dataset-A and 0.36 for Dataset-B under the influence of model imbalance. Previous study (28) has reported that, for datasets with an imbalance rate of approximately 25%, the baseline MCC is around 0.20. In contrast, our proposed model exhibited a comparable performance, achieving the lift of 1.8 to 2.4 over the mentioned baseline. This improvement indicates a moderate to strong predictive capability in addressing binary classification tasks under conditions of class imbalance (17-18%). Meanwhile, the MCC metric’s lift also evaluated based on our model’s prevalence and it is observed as approximately 2.1 to 2.7, further supporting its robustness. Collectively, these findings suggest that the developed model provides competitive predictive performance in imbalanced settings, with MCC and PR-AUC values confirming a statistically meaningful enhancement over the baseline expectations.

**Figure 2:**
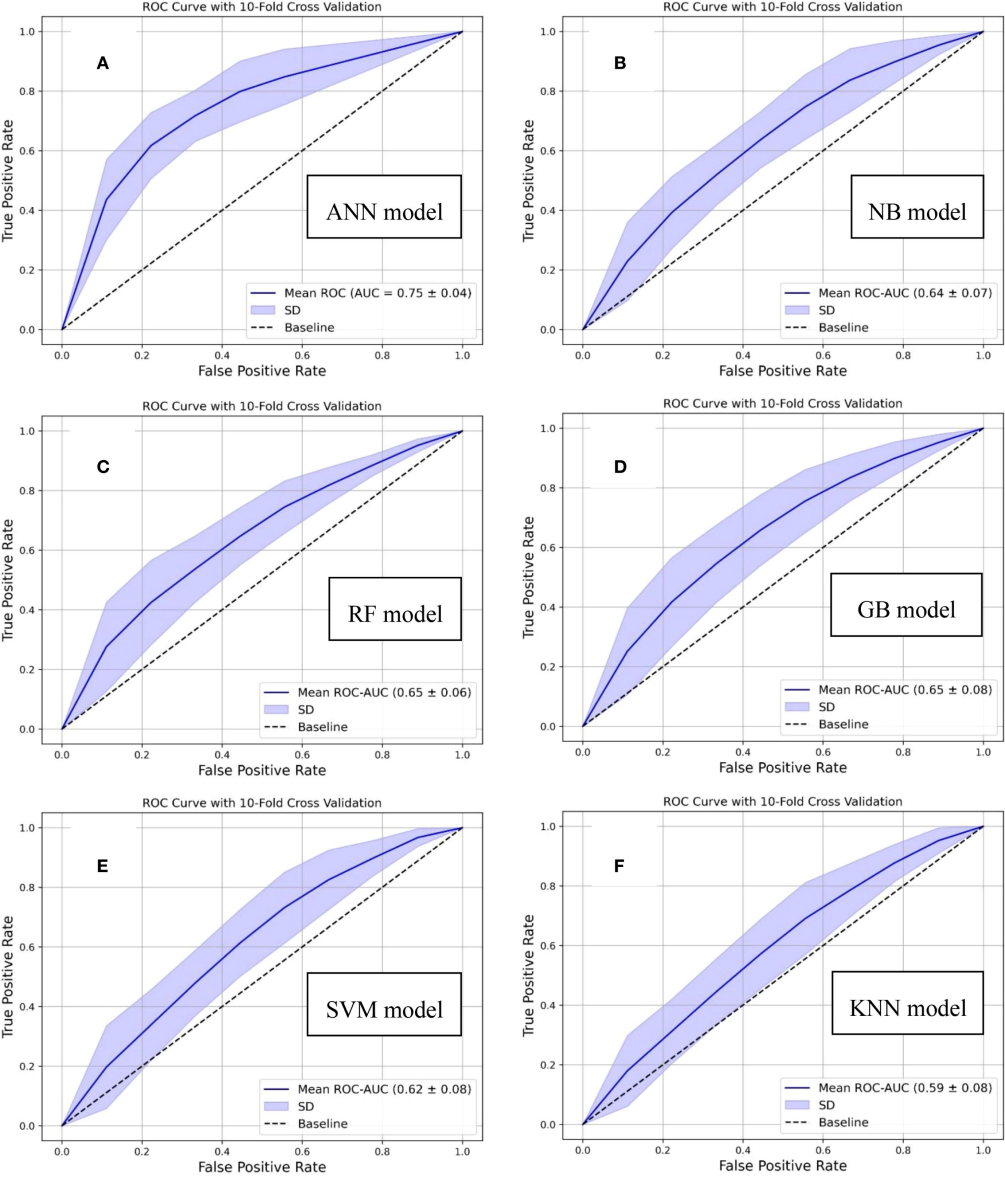
Comparative analysis based on ROC curves demonstrating the performance of conventional machine learning algorithms and our designed distant recurrence prediction framework: **(A)** Proposed artificial neural network (ANN)-based, **(B)** Naive bayes (NB)-based, **(C)** Random forest (RF)-based, **(D)** Gradient boost (GB)-based, **(E)** Support vector machine (SVM)-based, and **(F)** k-nearest neighbor (KNN)-based, predictive models.

Additionally, comprehensive comparative analyses were conducted to benchmark the proposed model against established conventional machine learning algorithms. This evaluation was carried out using the ROC curves as the primary performance metric along with the PR curves. The results of this comparative assessment are illustrated in **Figure 2** and **Supplementary Figure S3**, highlighting the relative effectiveness of each algorithm. In which the conventional machine learning models (Naive Bayes (NB), Random Forest (RF), Gradient Boosting (GB), Support Vector Machine (SVM), and K-nearest Neighbor (KNN)) achieved the ROC-AUC in the range of 0.59 to 0.65. From the comparative performance perspective, our designed ANN-based model demonstrated enhanced predictive capability, showing an improvement of 15.4% in ROC-AUC relative to traditional machine learning approaches. Additionally, statistical comparisons of the ROC-AUC values were conducted using the DeLong test. The results are summarized in **Supplementary File** (**Supplementary Table S1**), where each comparison pair the proposed predictive model with one of the existing models, evaluated against the true class labels. The DeLong test was implemented through the “MLstatkit” Python package. For each pairwise comparison, both the z-score and the corresponding p-value are evaluated. As shown in **Supplementary Table S1** (in **Supplementary File**), all p-values are observed below the statistical significance level (0.05), thereby indicating the difference between the two ROC-AUCs to be statistically significant. Furthermore, negative z-scores indicate the ROC-AUC of the proposed model is higher than other models.

### 3.2 Feature importance study

**Figure 3:**
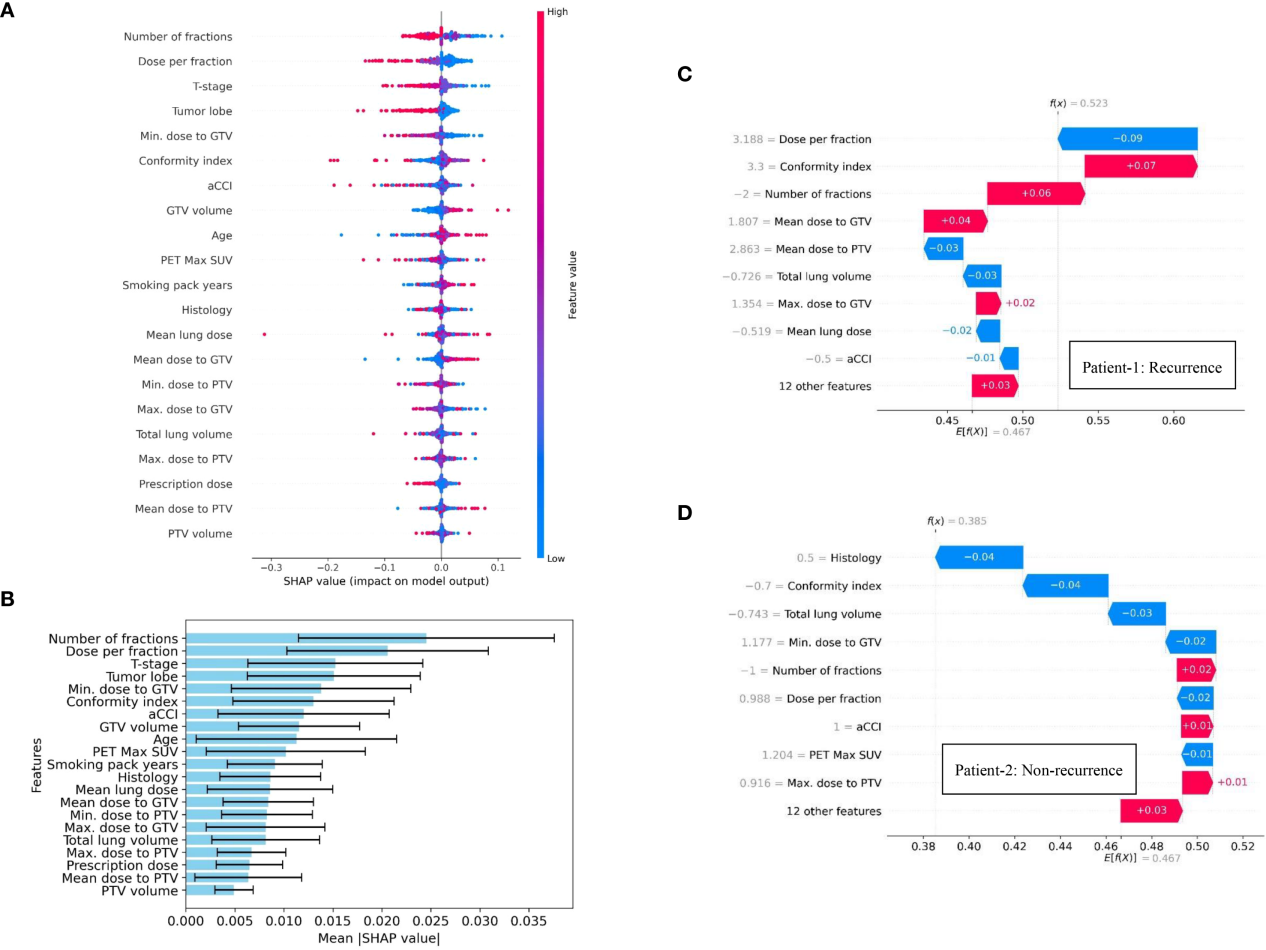
SHAP summary visualization, highlighting the contribution of individual dosimetric and clinical features on distant recurrence prediction in NSCLC patients treated with SBRT: **(A)** Beeswarm plot illustrating feature contribution to model’s predictive performance, **(B)** Feature importance ranking plot using 10-fold cross validation technique, and **(C, D)** SHAP force plots for two patients (Patient-1: recurrence & Patient-2: non-recurrence).

To enhance clinical interpretability of the developed ANN-based model for predicting distant recurrence, a SHAP framework was applied. The associations between treatment response following SBRT and the feature variables (dosiomic and clinical factors) used to construct the distant recurrence prediction model were evaluated using a SHAP-based approach. The SHAP summary (beeswarm) plot shown in **Figure 3A** depicts the relative contributions of all 21 features to the prediction of distant recurrence in early-stage NSCLC patients treated with SBRT. Features with a broader absolute distribution of SHAP values exert a stronger influence on the prediction outcome. In this model, Number of fractions, Dose per fraction, T-stage, Tumor lobe, Minimum dose to GTV, Conformity index, aCCI, GTV size, Age, and PET Max SUV were identified as the top ten contributing features, as illustrated in **Figures 3A, B**. This study also presents the SHAP force plot, which enables patient-specific interpretation. Each feature’s contribution to the prediction is visualized, where positive values increase the prediction of distant recurrence and negative values reduce it relative to a baseline. The baseline represents the mean SHAP value across all features. In **Figures 3C, D**, the length of each arrow indicates the strength of the contribution. Red arrows denote positive effects, while blue arrows represent negative effects. For Patient 1, the SHAP value exceeded the baseline, indicating a high likelihood of distant recurrence, with the feature arrows quantitatively representing their contributions to the prediction, as shown in **Figure 3C**. Conversely, Patient 2, who did not experience recurrence, was also evaluated (**Figure 3D**); in this case, the SHAP value was below the baseline, reflecting a lower likelihood of recurrence. It is to be noted that variables such as Dose per fraction, T-stage, and dose distribution to the GTV were among the top ten most influential features identified by our ANN-based predictive model, emphasizing their relevance in the context of distant recurrence prediction. However, the existing study (25) highlights that features like primary tumor dimension, and age did not rank within the top ten, suggesting a comparatively lower influence on the model’s predictive capacity in this specific clinical context. In contrast, our feature importance analysis identified both age and primary tumor dimension as belonging to the top ten contributing features, suggesting a more prominent role in predictive capacity within our model.

SHAP-based analysis addresses a key limitation of machine learning-based prediction models by providing the transparency or quantitative justification for the model’s prediction. In this study, SHAP-based approach identified the influential features which are aligns with the clinical knowledge. This transparency may enhance the clinician confidence in ANN-based prediction model and facilitate the integration into multidisciplinary decision-making. This insight could assist clinicians in refining the future treatment planning strategy while optimizing the dose scheduling. Moreover, SHAP summary plots identify T-stage as one of the most contributing features for our designed prediction model. Notably, higher T-stage, indicating larger or more locally advanced tumors, was often associated with a high likelihood of distant recurrence in our study. This may assist the clinicians to plan toward more intensive treatment approaches, closer monitoring, or the use of adjuvant therapies. Minimum dose to GTV was also identified as a key contributing factor. SHAP-based feature importance analysis indicates that lower minimum dose is associated with an increased probability of distant recurrence. Therefore, clinicians can use this insight to identify the underdosed tumor region and optimize the radiation plans accordingly. Thus, the developed prediction model while providing insights into key contributing features, may facilitate the early assessment of distant recurrence in NSCLC patients and enable the design of individual optimized treatment strategies for subsequent care followed by the initial course of therapy.

## 4 Discussions

In this study, an ANN-based deep learning model was developed to predict distant recurrence by integrating patient-specific clinical parameters and SBRT dose distribution characteristics. The primary aim of this study is to stratify early-stage NSCLC patients based on their likelihood of experiencing distant recurrence after SBRT, irrespective of the timing of the event. This problem is formulated as a binary classification problem, wherein the model is designed to predict the likelihood of distant recurrence while ensuring interpretability to facilitate clinical insight and decision-making. The innovative aspect of this study lies in the integration of diverse dose distribution characteristics commonly available clinical variables. Notably, the distribution of BED values in this study demonstrates a diverse dosing range compared to that reported in existing studies (21, 22). While existing literature predominantly focuses on clinical features in combination with only total prescribed dose and PTV or GTV sizes. The proposed model demonstrated superior predictive performance, reflecting a higher ROC-AUC when utilizing a comprehensive set of twenty-one input features, in comparison to traditional machine learning approaches. To ensure the robustness of the designed model, the predictive performance was also conducted on an independent dataset from a different institute. Furthermore, this study identified and highlighted the most influential features contributing to the ANN model’s predictive capacity for distant recurrence. Preliminary findings, as illustrated in **Tables 2** and **3**, highlight the emerging potential of predictive modeling to support clinical decision-making in the context of treatment planning for distant recurrence. This approach is especially pertinent to post-treatment outcomes following SBRT, a modality that is increasingly established as a standard of care for early-stage NSCLC patients who are unsuitable for surgical intervention. Moreover, this study employed a comprehensive set of performance metrics, including ROC-AUC, PR-AUC, sensitivity, specificity, weighted average F1-score, MCC, and positive predictive value, to evaluate the proposed model. In contrast, most existing studies on recurrence prediction have primarily reported ROC-AUC, with only one study also considering PR-AUC (24). Such limited evaluation may be insufficient in the context of low recurrence rates, where class imbalance poses a significant challenge. By incorporating a broader range of performance metrics, our study provides a more thorough assessment of model performance and strengthens the evidence for its predictive utility.

The key finding of this study is that integrating clinical and dosimetric features enhanced the predictive model’s performance. As shown in **Table 2**, the combined approach yielded a notable improvement in ROC-AUC, outperforming models that relied solely on either clinical or dosimetric factors by 6% and 9%, respectively. Furthermore, the integrated model demonstrated superior performance across multiple metrics. Specifically, PR-score improved by 39% over the clinical-only model and 15% over the dosiomic-only model; MCC increased by 41% and 26%, respectively; and positive predictive value rose by 42% and 29%, respectively. However, in the context of predicting distant recurrence among patients with early-stage NSCLC followed by SBRT, the development of prediction models based on dosimetric features remains relatively underexplored. Few previous studies have primarily focused on integrating selected clinical parameters with a limited set of dosiomic features to develop models for treatment response prediction (24, 25). For example, Mohamed et al. (24) utilized only patient-specific clinical features, including demographic, diagnostic, and biomarker data, to predict early disease recurrence in NSCLC patients. Their random forest-based model achieved an ROC-AUC of 0.68 (0.03) using five-fold cross-validation. In contrast, our model, which combines both dosimetric and clinical features, demonstrated improved performance, with ROC-AUC increases of 10% on the internal dataset and 4% on the external dataset. Another existing study by Hindocha et al. (25), incorporated additional treatment-related parameters, including total radiation dose, PTV size, number of fractions, and treatment modality along with demographic and clinical factors, in developing predictive models for recurrence, recurrence-free survival, and overall survival outcomes. In their study, the highest predictive performance for recurrence was achieved using KNN and RF models, yielding a ROC-AUC of 0.68 for internal validation and 0.72 for external validation. However, the analysis did not specify the subtype of recurrence being predicted, and the study cohort comprised patients treated with various treatment approaches, including chemoradiotherapy, SBRT, and conventional radiotherapy.

Several studies also explored the radiomic-based imaging features to develop the recurrence prediction model (16, 17, 19–23). Nonetheless, the existing literature reveals limited studies (19–21) specifically addressing the prediction of distant recurrence following SBRT in patients with early-stage NSCLC. An existing study by Coroller et al. (19) reported a C-index of 0.60 for their developed multivariable Cox-regression model to predict distant recurrence, while integrating clinical variables and planning CT-image-based radiomic features. This performance is comparatively lower than that of our model. Furthermore, Lafata et al. (20) also explored the relationship between radiomic features extracted from pre-treatment X-ray CT scans and clinical outcomes in NSCLC patients undergoing SBRT. Utilizing a multivariable logistic regression framework, they assessed the model’s ability to distinguish between non-recurrence, local recurrence, and non-local recurrence, based on 70 NSCLC patients’ data from a single institution. Notably, their designed model achieved the ROC-AUC of 0.60 (0.04) for predicting non-local recurrence, encompassing both regional and distant recurrence Meanwhile, our developed model achieved superior predictive performance with the improvements of 25% on Dataset-A and 18% on Dataset-B compared to the other results (20). Similarly, Nemoto et al. (21) reported that their SVM model, trained on the ten most important radiomic features from pre-treatment PET and CT images of 82 NSCLC patients, achieved ROC-AUC values of 0.64 for CT-based features, and 0.60 for PET-based features in predicting recurrence after SBRT. In contrast, our designed model based on dosiomic and clinical features exhibited relative superior performance while assessing a diverse set of performance metrics, as shown in **Table 2** and **3**. Notably, heterogeneity in imaging protocols across diverse clinical settings poses substantial challenges to the standardization of radiomics-based predictive models. This heterogeneity can degrade the reproducibility and comparability of model outcomes. Furthermore, in busy clinical environments, the complexity and large number of radiomics features may hinder interpretability for clinicians, potentially reducing the efficiency and effectiveness of clinical decision-making.

In contrast to prior studies on distant recurrence prediction using machine learning models, the strength of our investigation was conducted using data from two independent institutions and included a substantially larger cohort of early-stage NSCLC patients treated with SBRT only. The proposed model based on dosiomic and clinical features demonstrated superior predictive performance in comparison to existing studies on predicting distant recurrence (20, 21, 24, 25). To the best of our knowledge, the existing literature on distant recurrence prediction in early-stage NSCLC patients undergoing SBRT has not comprehensively explored the integration of heterogeneous dose distribution parameters alongside clinically relevant features in the development of machine learning-based predictive models. Furthermore, the application of SHAP in this study facilitated the interpretation of feature contributions, enabling the identification of the input variables that most significantly influenced the predictive performance of the designed ANN model.

This study has certain limitations related to treatment heterogeneity, as the model was specifically developed for patients with early-stage NSCLC (stage I-II) undergoing SBRT. Obtaining a sufficiently large dataset from a single institution posed challenges; therefore, data from two institutions were included in this retrospective analysis. Nonetheless, to enhance the generalizability and robustness of the proposed model, future investigations should incorporate larger patient cohorts and data from multiple institutions. Another limitation of this study is the imbalance rate of distant recurrence events. According to existing literature (29), the recurrence rate is approximately 30%, and such an imbalance can substantially influence model performance. To more effectively demonstrate the utility of the proposed model, additional data from patients with recurrence are needed to capture broader patterns and improve predictive accuracy. Despite this limitation, our model, with an approximately 15% imbalance rate, achieved significantly enhanced performance by applying class weighting and focal loss; however, the imbalance still had an impact on PR-AUC and MCC values. In addition, factors beyond dosimetric and clinical variables, such as pathological and genomic data, may also contribute to distant recurrence prediction, but these were not available within the constraints of our dataset. Future work will therefore focus on incorporating a broader spectrum of features, including pathological and genomic information. The promising findings of this study may serve as a foundation for developing future models that are more specific for a diverse set of dose data and clinical factors. Several studies (22, 30–33) have emphasized the prediction of distant recurrence survival risk. In this context, evaluating temporal drift and the stability of predictive performance across different time windows is crucial. This represents an important avenue for future research, and subsequent studies will aim to extend our framework to include temporal split sensitivity analyses. Accordingly, future work will also focus on adapting our model to survival-based approaches to capture time-dependent risk.

## 5 Conclusions

This study demonstrates the design and external validation of an ANN-based distant recurrence prediction model for early-stage NSCLC patients treated with SBRT. This study also emphasizes the influence of combination of diverse dosiomic and clinical features that significantly enhance the model’s predictive performance while involving a detailed SHAP-based feature importance analysis. These findings underscore the model’s enhanced performance, highlighting its potential as a supportive tool for clinicians in optimizing personalized and effective radiation therapy strategies. However, investigation into model’s impact on assisting in clinical decision-making process will be essential to assess its practicability in real-world clinical settings.

## Data Availability

The original contributions presented in the study are included in the article/**Supplementary Material**. Further inquiries can be directed to the corresponding author.
