# Supplementary material for: Artificial neural network-augmented dosiomic integration for predicting distant recurrence in NSCLC patients treated with SBRT
Source: Front Oncol. 2025 Sep 25;15:1669954. doi: 10.3389/fonc.2025.1669954 (PMC12507626; doi:10.3389/fonc.2025.1669954)
Supplement: Supplementary file 1 [file DataSheet1.pdf]

Supplementary Material

1 Designed ANN-based Distant Recurrence Prediction Model's Architecture

```
model.summary()
```

Model: "sequential_9"

Layer (type)	Output Shape	Param #
dense_90 (Dense)	(None, 50)	1,100
batch_normalization_81 (BatchNormalization)	(None, 50)	200
dense_91 (Dense)	(None, 100)	5,100
batch_normalization_82 (BatchNormalization)	(None, 100)	400
dense_92 (Dense)	(None, 500)	50,500
batch_normalization_83 (BatchNormalization)	(None, 500)	2,000
alpha_dropout_45 (AlphaDropout)	(None, 500)	0
dense_93 (Dense)	(None, 350)	175,350
batch_normalization_84 (BatchNormalization)	(None, 350)	1,400
alpha_dropout_46 (AlphaDropout)	(None, 350)	0
dense_94 (Dense)	(None, 250)	87,750
batch_normalization_85 (BatchNormalization)	(None, 250)	1,000
alpha_dropout_47 (AlphaDropout)	(None, 250)	0
dense_95 (Dense)	(None, 150)	37,650
batch_normalization_86 (BatchNormalization)	(None, 150)	600
alpha_dropout_48 (AlphaDropout)	(None, 150)	0
dense_96 (Dense)	(None, 100)	15,100
batch_normalization_87 (BatchNormalization)	(None, 100)	400
alpha_dropout_49 (AlphaDropout)	(None, 100)	0
dense_97 (Dense)	(None, 50)	5,050
batch_normalization_88 (BatchNormalization)	(None, 50)	200
dropout_9 (Dropout)	(None, 50)	0
dense_98 (Dense)	(None, 30)	1,530
batch_normalization_89 (BatchNormalization)	(None, 30)	120
dense_99 (Dense)	(None, 1)	31

Total params: 1,150,125 (4.39 MB)
Trainable params: 382,321 (1.46 MB)
Non-trainable params: 3,160 (12.34 KB)
Optimizer params: 764,644 (2.92 MB)

Figure S1. Pipeline of the designed ANN-based distant recurrence model.

2 Algorithm of the Designed Prediction Model (Pseudo code)

Algorithm: Distant Recurrence Prediction Model based on ANN architecture

Load Libraries: Import Numpy, Pandas, Matplotlib, Tensorflow, Keras
Load and Preprocess Dataset:
- Load the internal dataset
- Data normalization using RobustScaler() function

Initialization:
- Define number of input features
- Define number of hidden layers
- Define number of neurons for hidden layers
- Define activation functions
- Define the number of splits for cross-validation (N_s)

Main cross-validation loop:
For each *train_indices, test_indices* **in** cross-validation split (N_s):
Select ***X_train*** using train_indices
Select ***X_test*** using test_indices
Select ***Y_train*** using train_indices
Select ***Y_test*** using test_indices

Training and Threshold tunning split:
X_train_1, ***Y_train_1***, ***X_validation_1***, ***Y_validation_1*** = train_test_split(***X_train***, ***Y_train***, test_size=0.30, random_state=42)

Development of ANN architecture (***Model***):
- Layer-1: Input layer (size= number of input features)
- Layer-2: First Hidden Layer (size=50, Activation function=ReLU, L2 regularizer(0.01))
- Layer-3: Batch Normalization (penalty coefficient of 0.01)
- Layer-4: Second Hidden Layer (size=100, Activation function=ReLU, L2 regularizer(0.01))
- Layer-5: Batch Normalization (penalty coefficient of 0.01)
- Layer-6: Third Hidden Layer (size=500, Activation function=ReLU, L2 regularizer(0.01))
- Layer-7: Batch Normalization (penalty coefficient of 0.01)
- Layer-8: Fourth Hidden Layer (size=350, Activation function=ReLU, L2 regularizer(0.01))
- Layer-9: Batch Normalization (penalty coefficient of 0.01)
- Layer-10: Fifth Hidden Layer (size=250, Activation function=ReLU, L2 regularizer(0.01))
- Layer-11: Batch Normalization (penalty coefficient of 0.01)
- Layer-12: Sixth Hidden Layer (size=150, Activation function=ReLU, L2 regularizer(0.01))
- Layer-13: Batch Normalization (penalty coefficient of 0.01)
- Layer-14: Seventh Hidden Layer (size=100, Activation function=ReLU, L2 regularizer(0.01))
- Layer-15: Batch Normalization (penalty coefficient of 0.01)
- Layer-16: Sixth Hidden Layer (size=50, Activation function=ReLU, L2 regularizer(0.01))
- Layer-17: Batch Normalization (penalty coefficient of 0.01)
- Layer-18: Eight Hidden Layer (size=30, Activation function=ReLU, L2 regularizer(0.01))
- Layer-19: Batch Normalization (penalty coefficient of 0.01)
- Layer-20: Output Layer (size= 1, Activation function=Sigmoid)

Compile the ANN model with ADAM optimizer with focal loss function and class weighting strategies
Train the model with *(**X_train_1**, **Y_train_1**)* with early stopping criteria
Model*.fit(**X_train_1**, **Y_train_1**, epochs=100, validation_data=(**X_validation_1**, **Y_validation_1**), batch_size=16, class_weight=class_weight, callbacks=[early_stopping])*

Threshold optimizing:
Y_validation_predict = ***Model***.predict(***X_validation_1***)
*Precision, Recall, Thresholds = precision_recall_curve(**Y_validation_1**, **Y_validation_predict**)*

Evaluate the F1-score: *f1_scores* = Harmonic mean of *Precision and Recall*
Find the Threshold for maximum F1-score:
Best Index = np.argmax(*f1_scores*)
Best_Threshold = *Thresholds* [*Best Index*]

Validation on test data (***X_test***, ***Y_test***):
- Predict the probability
- Perform binary classification based on the optimal threshold (*Best_Threshold)*
- Generate the binary classification (**recurrence or non-recurrence**)

Model Performance Evaluation: using Sklearn.metric library
Generation of the ROC curve using Matplotlib library
Generation of the PR curve using Matplotlib library
Save the best ***Model*** for external validation
Evaluate the Performance Metrics: ROC-AUC, PR-AUC, Sensitivity, Specificity, Weighted average F1-score, MCC, Positive predictive value

External validation:
Load the dataset
Normalized using previously normalized function used for internal dataset
Initialize the Number of Bootstrap Samples
For each iteration **in** Number of Bootstrap Samples:
Determine the random indices
Select ***X_bootstrap*** using indices
Select ***Y_bootstrap*** using indices
Call the save trained and validated model (***Model***)
Predict the recurrence using (***X_bootstrap***, ***Y_bootstrap****)*
Model Performance Evaluation: using Sklearn.metric library
Generation of the ROC curve using Matplotlib library
Generation of the PR curve using Matplotlib library
Evaluate the Performance Metrics: ROC-AUC, PR-AUC, Sensitivity, Specificity, Weighted average F1-score, MCC, Positive predictive value

---

3 Python Version and Software Stack

In this study, to perform the simulations Python 3.10 within the Jupyter Notebook environment was utilized. Python code was built using following libraries: TensorFlow (version 2.17.0) and Keras (sequential API, version 3.4.1), NumPy (version 1.26.4) for numerical computations, Pandas (version 2.2.2) for data preprocessing, Matplotlib (version 3.8.4), and Scikit-learn (version 1.4.2) for data splitting, model performance evaluations, comparison analysis with conventional models (such as Support Vector Machine, Decision Tree, K-Nearest Neighbor, Gradient boosting, Naive Bayes, and Random Forest). All algorithms were executed on a Windows 11 Enterprise system equipped with an Intel Core-i7-14700 CPU (2.10 GHz) and an NVIDIA GeForce RTX 4070 GPU.

4 Feature Distribution Across Two Datasets and Additional Results

Source / extraction method: For SBRT radiotherapy (RT) planning, CT scan was performed using SOMATOM go-Open Pro CT-Sim system (Siemens, Malvern, PA, USA) with 1.0mm slice thickness. The radiation oncologists used planning CT with lung window/level and PET/CT for delineating the target/tumor volume (gross tumor volume (GTV)). The physicians incorporated appropriate margin to GTV for creating planning target volume (PTV). The organs at risk (OARs) were contoured by the dosimetrists using planning CT scan with organ/anatomy specific window/level settings; then all the contours were reviewed by the radiation oncologists and the physicists All the contours and the and RT plans were created using Eclipse treatment planning

system (version 16.1, Varian Medical System, Palo Alto, CA, USA) and Eclipse's dose volume histogram (DVH) was used for collecting require dosimetry data for this study. Patients' clinic data and information were gleaned from Electronic Medical/Health Record (EMR/EHR), Epic EHR (HYPERSPACE, Verona, WI, USA).

Figure S2. Feature distribution plot across two datasets (Dataset-A and Dataset-B)

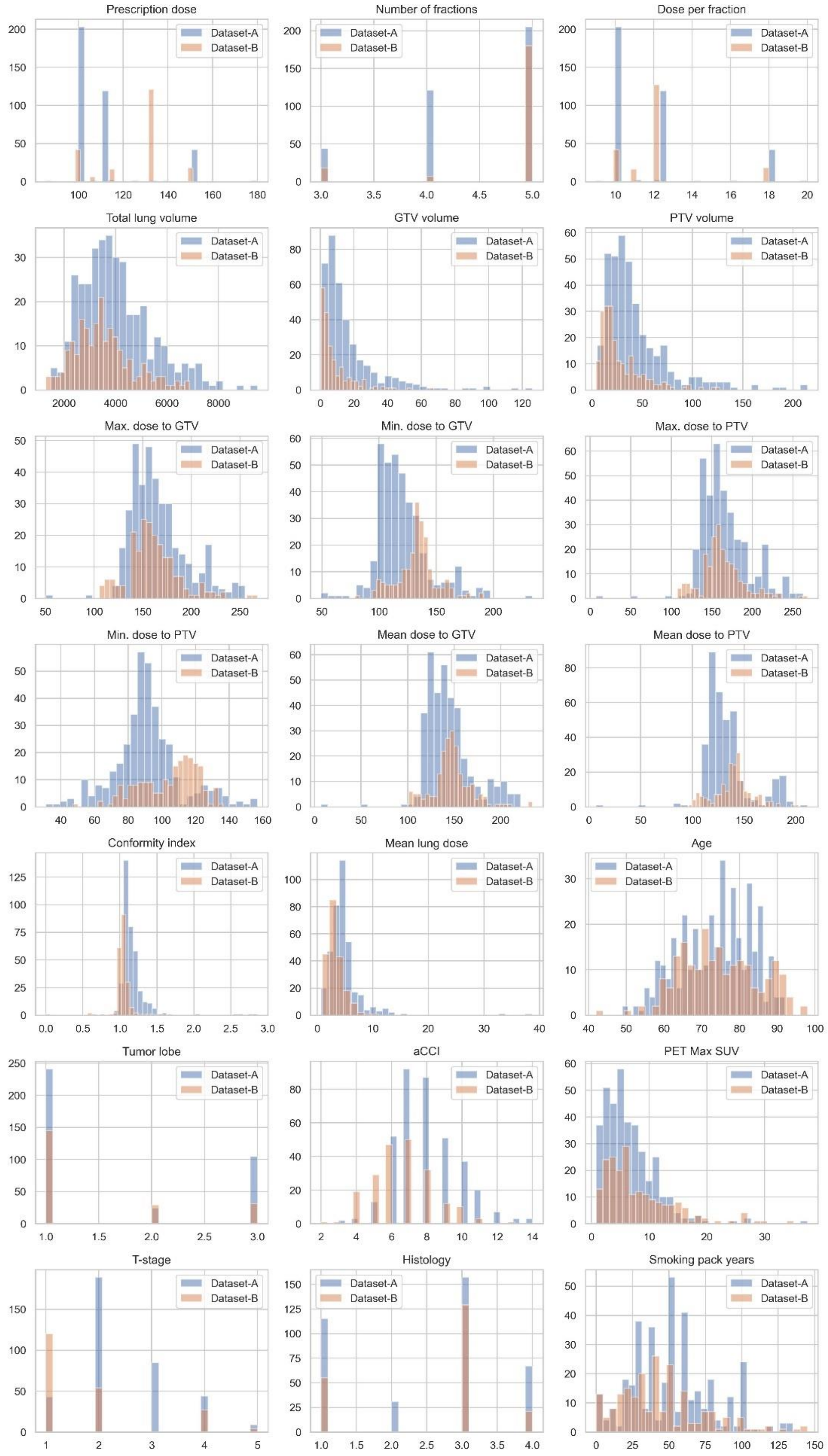

Tumor lobe→

1: Upper

2: Middle

3: Lower

Histology→

1: Adenocarcinoma

2: Non-small cell carcinoma

3: Squamous cell carcinoma

4: Others

T-stage→

1: T1a

2: T1b

3:T1c

4: T2a

5: T2b

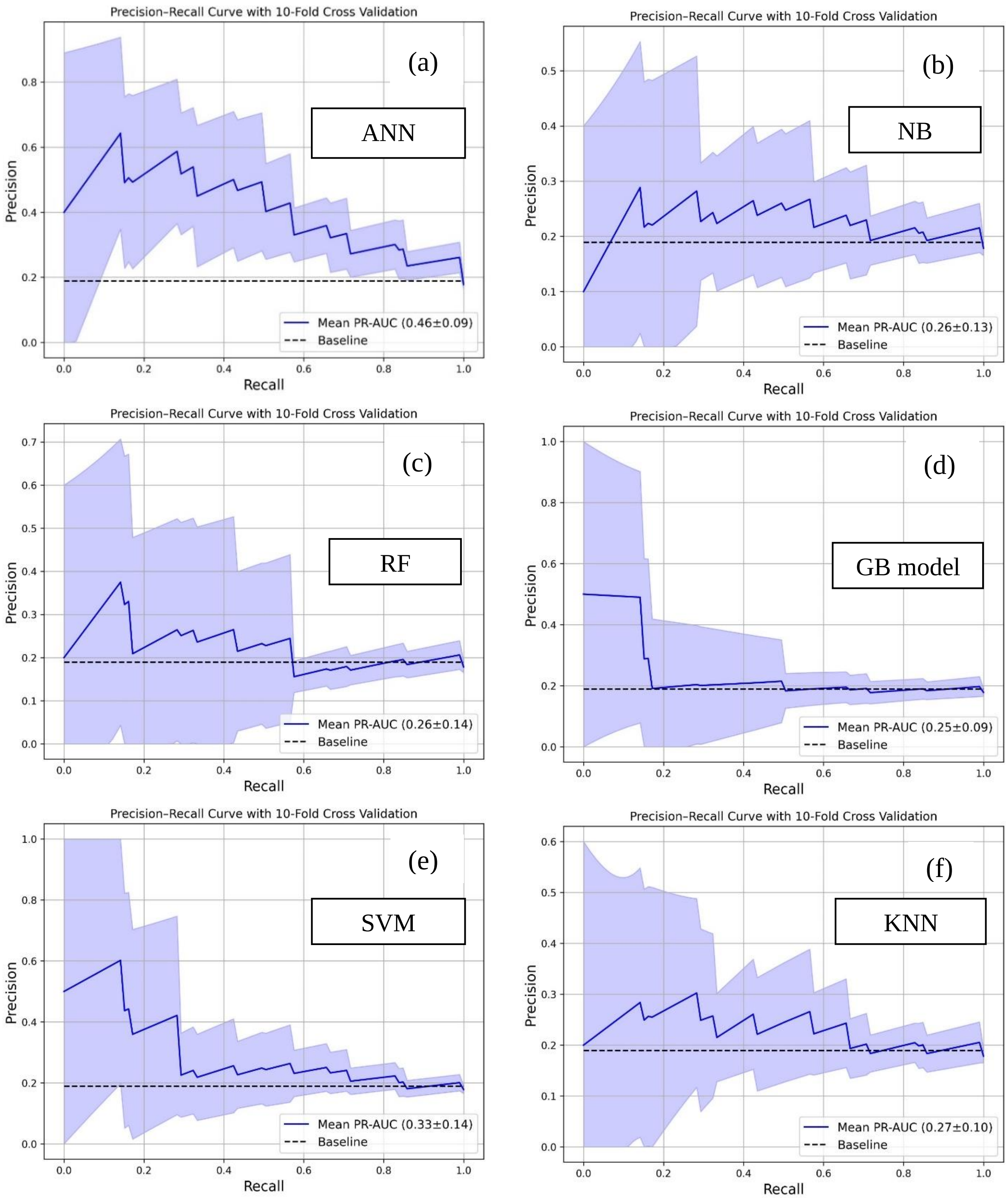

Figure S3: Comparative analysis based on Precision-Recall (PR) curves demonstrating the performance of conventional machine learning algorithms and our designed distant recurrence prediction framework: (a) Proposed Artificial Neural Network (ANN)-based, (b) Naive Bayes (NB)-based, (c) Random Forest (RF)-based, (d) Gradient Boost (GB)-based, (e) Support Vector Machine (SVM)-based, and (f) K-Nearest Neighbor (KNN)-based, predictive models.

Table S1: Performance analysis of DeLong tests for ROC-AUC differences between the proposed prediction model and the existing conventional models.

Model pair	z-score	p-value
Proposed vs NB-based model	-2.310	0.021
Proposed vs RF-based model	-2.210	0.027
Proposed vs GB-based model	-3.033	0.002
Proposed vs SVM-based model	-3.152	0.001
Proposed vs KNN-based model	-2.858	0.004
